# Supplementary material for: Regulation of striatal cells and goal-directed behavior by cerebellar outputs
Source: Nat Commun. 2018 Aug 7;9:3133. doi: 10.1038/s41467-018-05565-y (PMC6081479; doi:10.1038/s41467-018-05565-y)
Supplement: Supplementary file 1 — Supplementary Information [file 41467_2018_5565_MOESM1_ESM.pdf]

Supplementary Material

Regulation of striatal cells and goal-directed behavior by cerebellar outputs

Le Xiao, Caroline Bormann, Laetitia Hatstatt-Burklé, Peter Scheiffele

Biozentrum, University of Basel, 4056 Basel, Switzerland

Supplementary Figure 1

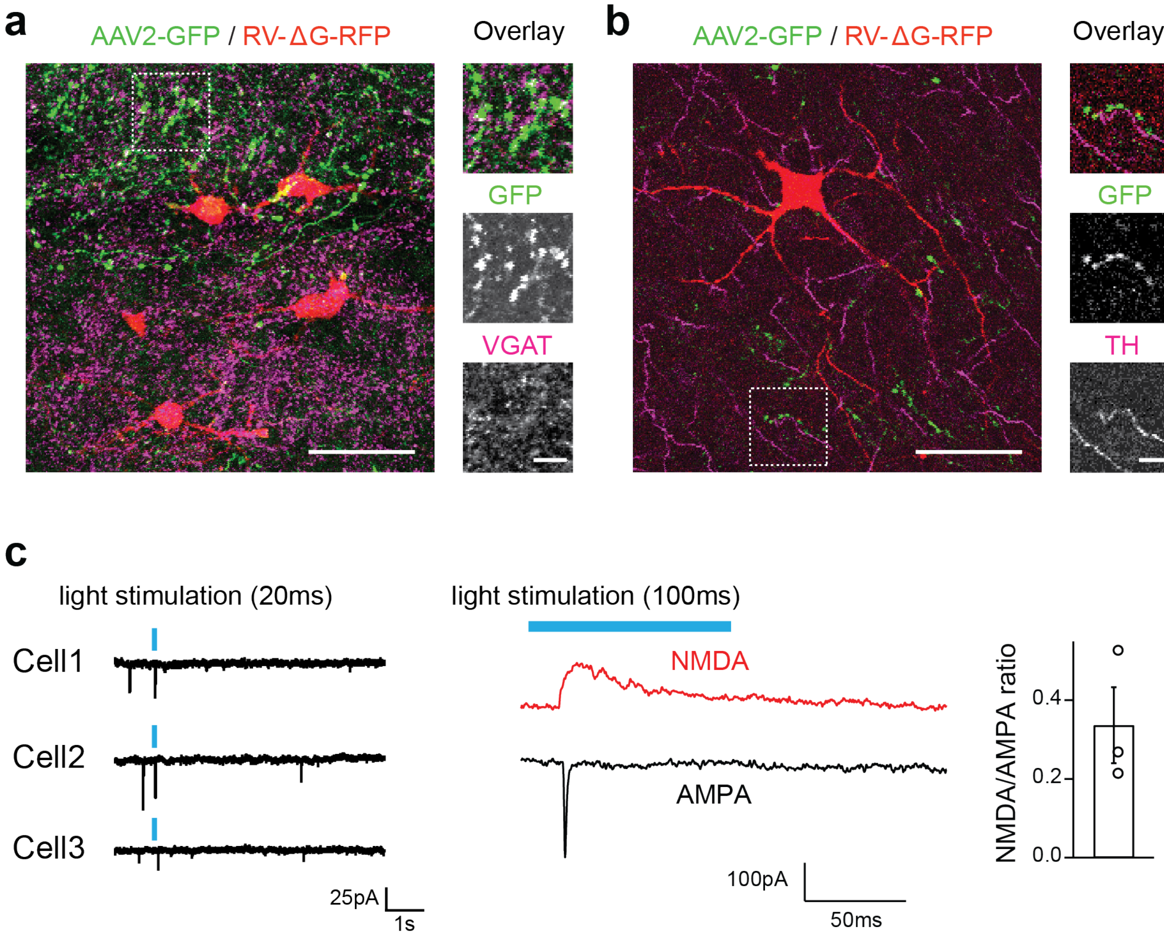

Supplementary Figure 1. DCN-derived axons in the ILN.

a, Stereotaxic injections of AAV2-GFP and RV- ΔG-RFP into DCN and dorsal striatum as described in Figure 1. Example image and magnified views from the parafascicular nucleus of injected mice stained for the inhibitory synapse marker vesicular GABA transporter (vGAT, magenta), GFP expressed in DCN axons and thalamic neurons retrogradely labeled with from the dorsal striatum with RV- ΔG-RFP. Scale bars: 50μm and 10μm, respectively.

b, As in ***a*** but labeled for the dopaminergic neuron marker tyrosine-hydroxylase (TH, magenta). Scale bars are as in a.

c, To assess function of DCN-ILN neuron connections ChR2-GFP was expressed in DCN neurons (stereotaxic injection of AAV2-ChR2-GFP as in Figure 1) and light-evoked currents were recorded from PaF neurons located in proximity of GFP-positive axons in acute slice preparations (postnatal day ~P35, 2-3 weeks after viral injection). Example EPSC traces from three cells derived from 3 different animals. The blue mark indicates the timing of a 20 ms light pulse (470nm). The right panel shows example EPSC traces elicited by 100 ms light pulse (470nm) recorded at +40mV (red) and -70mV (black) used to assess NMDA- to AMPA-receptor ratios (displayed as mean ± SEM from three PaF cells responding to ChR2-mediated stimulation).

Supplementary Figure 2

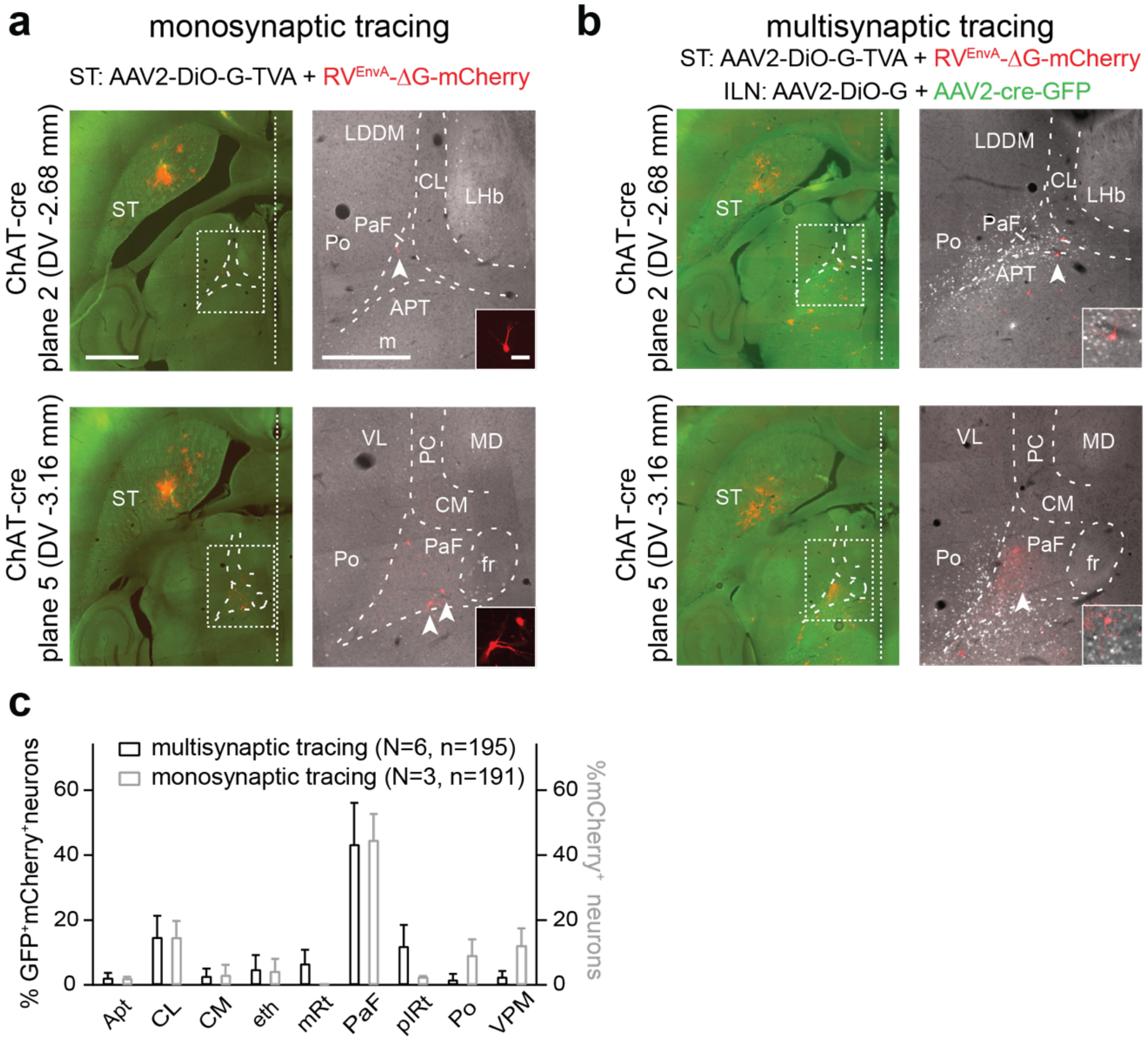

Supplementary Figure 2. Monosynaptic and multisynaptic viral tracing from ChAT-positive interneurons in the dorsal striatum.

a, Viral tracing was initiated from ChAT-cre positive cells by cre-dependent expression of the TVA receptor (AAV2-DiO-G-TVA) delivered by stereotaxic injection into the dorsal striatum of ChAT-cre mice at postnatal day 11-12, followed by injection of glycoprotein-deficient rabies viruses pseudotyped with the EnvA envelope protein at postnatal day 18-19. The left panels show mCherry signal (red) derived from the rabies virus at start sites in the dorsal striatum of ChAT-cre mice. The green signal is tissue autofluorescence shown to clarify the anatomy. The dashed area is enlarged in the right panel to visualize transsynaptically labeled ILN neurons at two dorsal-ventral planes (red cells, plane 2 and 5). Example ILN neurons pointed by error heads are shown in insets (scale bars: 1mm, 500µm and 50µm).

b, Multisynaptic tracing configuration: As in ***a*** but with an additional injection of AAV2-DiO-G and AAV2-cre-GFP into the intralaminar nuclei. Expression of cre-GFP is shown in green (left)

and white in the enlargement (right). GFP-positive cells are detected in CL, PaF, Po and APT. By contrast, little of no expression of GFP signal is seen in LHb, LDDM or VL. Example ILN neurons pointed by error heads are shown in insets (scale bar, 50μm).

c, Quantitative comparison of thalamic cells marked by mono- versus multisynaptic tracing. For monosynaptic tracing, the relative distribution of mCherry-positive cells across thalamic nuclei was assessed (N=3 ChAT-cre mice, total of n=191 cells, displayed in gray). For multisynaptic labeling GFP/mCherry double-positive cells were quantified (N=6 ChAT-cre mice, n=195 cells, displayed in black). The plots show means ± SEM.

Supplementary Figure 3

a

Center of injection site ±SEM (mm)

	D1R-cre		A2A-cre		ChAT-cre	
AP	0.09	0.08	0.07	0.10	0.03	0.05
LM	2.10	0.22	2.00	0.04	2.03	0.05
DV	2.20	0.12	2.37	0.08	2.28	0.06

b

dimensions of injection site ±SEM (mm)

	D1R-cre		A2A-cre		ChAT-cre	
AP	1.91	0.33	2.01	0.29	1.64	0.27
LM	1.77	0.12	1.79	0.19	1.54	0.15
DV	1.35	0.19	1.63	0.17	1.38	0.10

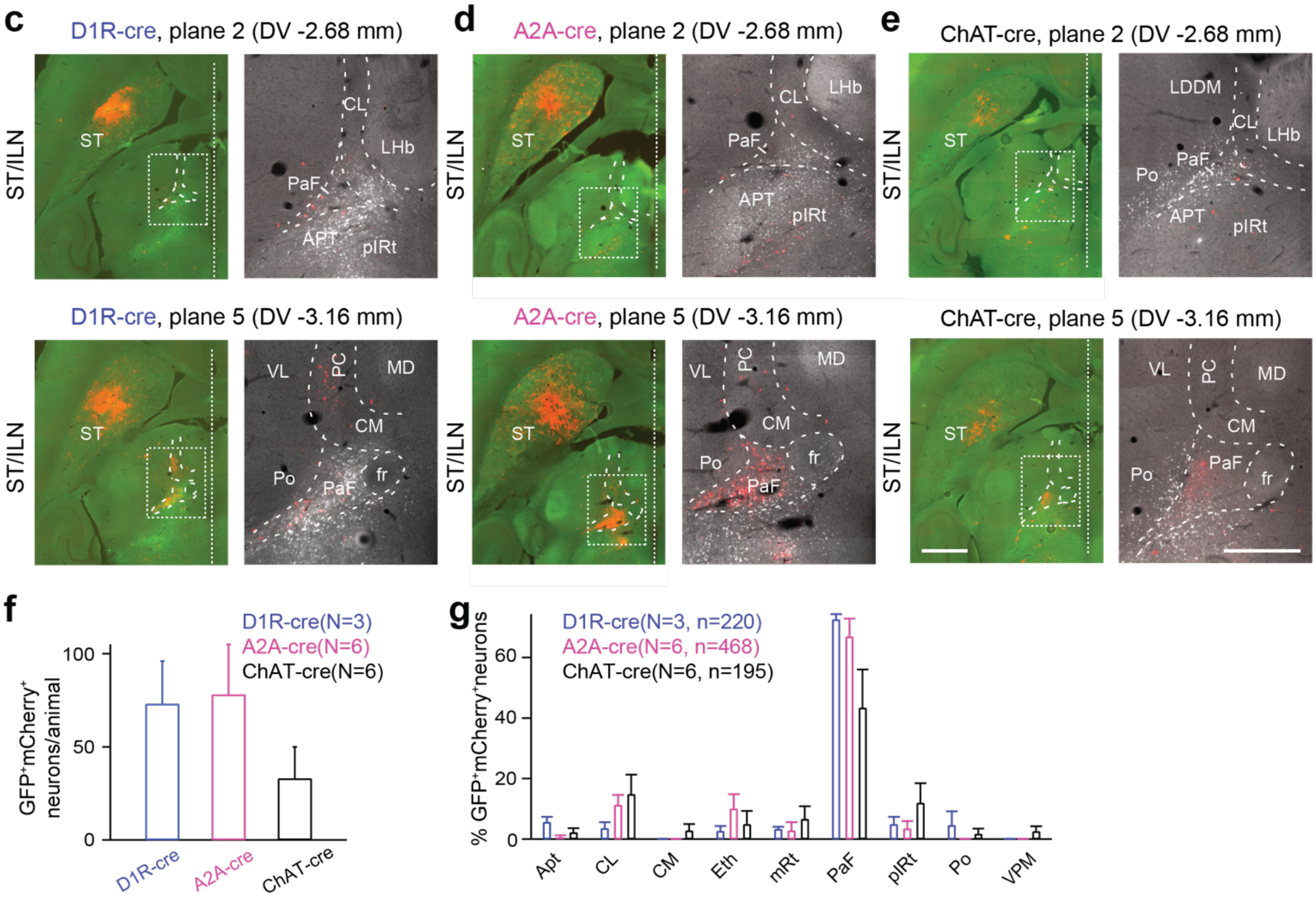

Supplementary Figure 3. Retrograde multisynaptic tracing from dorsal striatum of D1R-cre, A2A-cre and ChAT-cre mice.

a, Center of the viral injection sites for retrograde rabies tracing experiments in Fig. 3. The anterior-posterior (AP) lateral-medial (LM) and dorso-ventral (DV) coordinates of the mCherry-positive infection site for mice analyzed in Fig. 3 (see the injection scheme in Fig. 3a).

b, Dimensions of the infected areas in the dorsal striatum were quantified. The primary infected areas cover the anterior dorsal-lateral striatum as well as the posterior dorsal-medial striatum. The average primary infected areas for D1R- and A2A-cre mice was slightly larger than for ChAT-cre mice (presumably due to the higher abundance of MSNs).

c, mCherry (red) and GFP signal (green) derived from the rabies virus at start sites in the dorsal striatum and AAV2-cre-GFP injection into the ILN of D1R-cre mice and trans-synaptically labeled ILN relay sites at two dorsal-ventral planes (plane 2 and 5). Left image

shows an overview, right image with an enlargement of the dashed ILN area with the red mCherry signal overlaid on GFP signal, now represented in white.

d, as in ***c,*** but for A2A-cre mice.

e, as in ***c,*** but ChAT-cre mice. Scale bars: 1mm and 500μm, respectively.

f, Average number of GFP and mCherry double-positive thalamic cells retrogradely labeled from the dorsal striatum in D1R-cre, A2A-cre, and ChAT-cre mice. Mean ± SEM. N=3 D1R-cre mice, N=6 A2A-cre mice, N=6 ChAT-cre mice.

g, Relative distribution of GFP and mCherry double-positive cells (which represent the candidate relay neurons) across thalamic nuclei (N= 3 mice and n=220 neurons for D1R-cre mice, N=6 mice and n=468 neurons for A2A-cre, and N=6 mice and n=195 neurons for ChAT-cre mice). Note that the numbers plotted for ChAT-cre mice are the same as displayed in the comparison of mono- and multisynaptic labeling in Supplementary Figure 2c. Graphs display mean±SEM.

Supplementary Figure 4

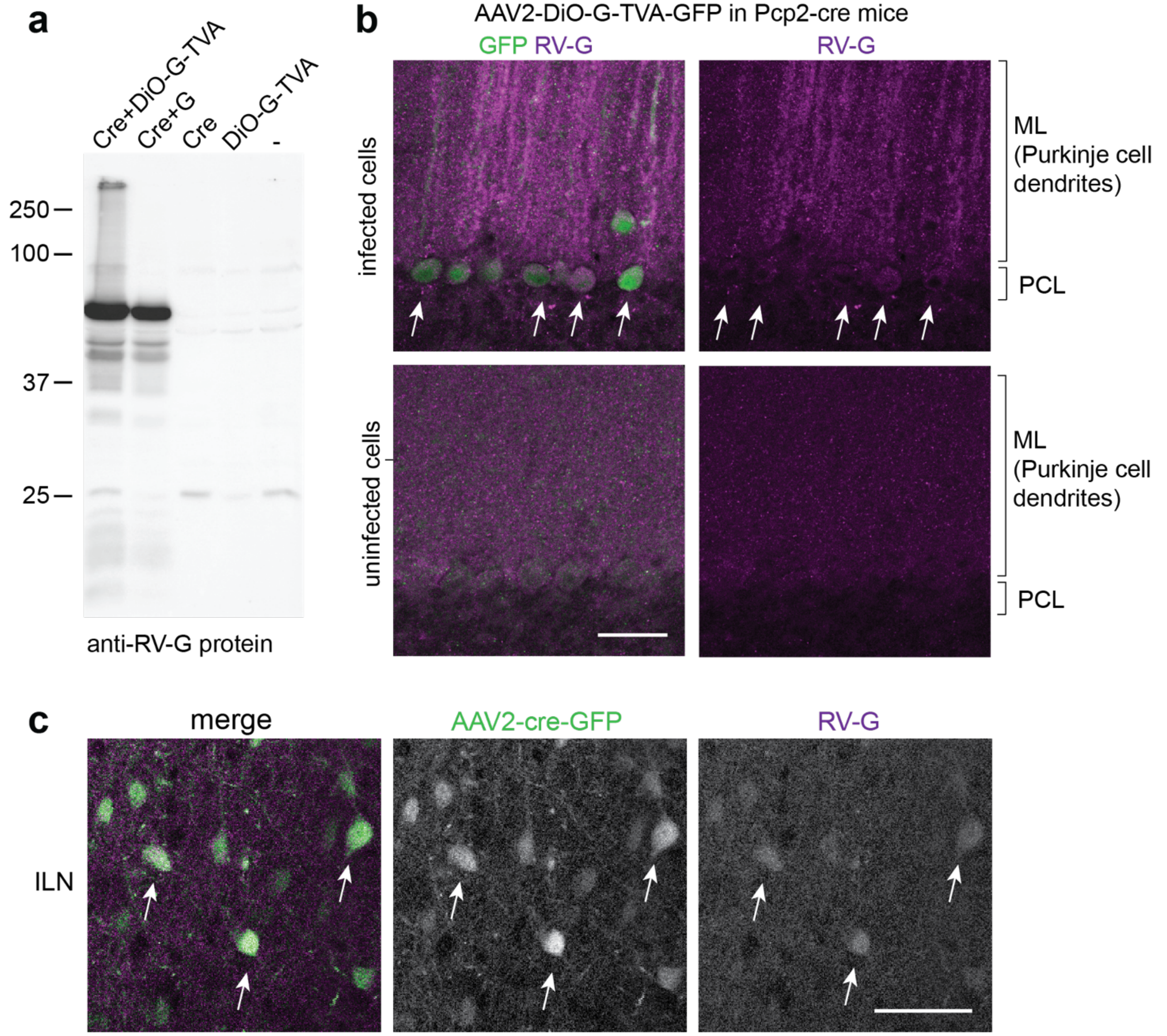

Supplementary Figure 4. Generation of a polyclonal rabies virus G protein antibody.
a, Western blot of HEK293T cells transfected with expression vectors for combinations of cre-recombinase, cre-dependent rabies G protein (DiO-G-TVA-GFP), or cre-independent rabies G protein (G). Non-transfected cells (-) serve as a negative control. The blot was probed with guinea pig anti-rabies glycoprotein antibodies (anti-RV-G). Molecular weight markers are indicated on the left in kDa.
b, Detection of rabies G protein in mouse cerebellum. AAV2-DiO-G-TVA-GFP viruses were injected into the cerebellar cortex of Pcp2-cre mice (expressing cre-recombinase selectively in Purkinje cells, N=3). The panels show confocal microscopy images of immunohistochemistry with guinea pig anti-RV-G and rabbit anti-GFP antibodies. Anti-RV-G antibodies label the Purkinje cell dendrites in the molecular layer (ML). As a negative control in lower two panels,

part of the cerebellar cortex away from the viral injection site imaged with identical confocal settings (gain, offset, laser power) are shown. Scale bar: 100µm.

c, Detection of rabies G protein in relay cells in the mouse intralaminar nucleus. The image shows cells in the PaF of mice were viral G protein was expressed using co-injection of AAV2-DiO-G and AAV2-cre (as used for multisynaptic tracing in Figure 3). The middle panel shows anti-GFP immunoreactivity and right panel shows anti-RV-G immunoreactivity. Some coexpressing cells are marked with arrows. Quantitative assessment indicated that most but not all GFP-positive cells are also immunoreactive for the anti-G antibody (82.5% of 714 cells from N=2 mice). Scale bar: 200µm.

Supplementary Figure 5

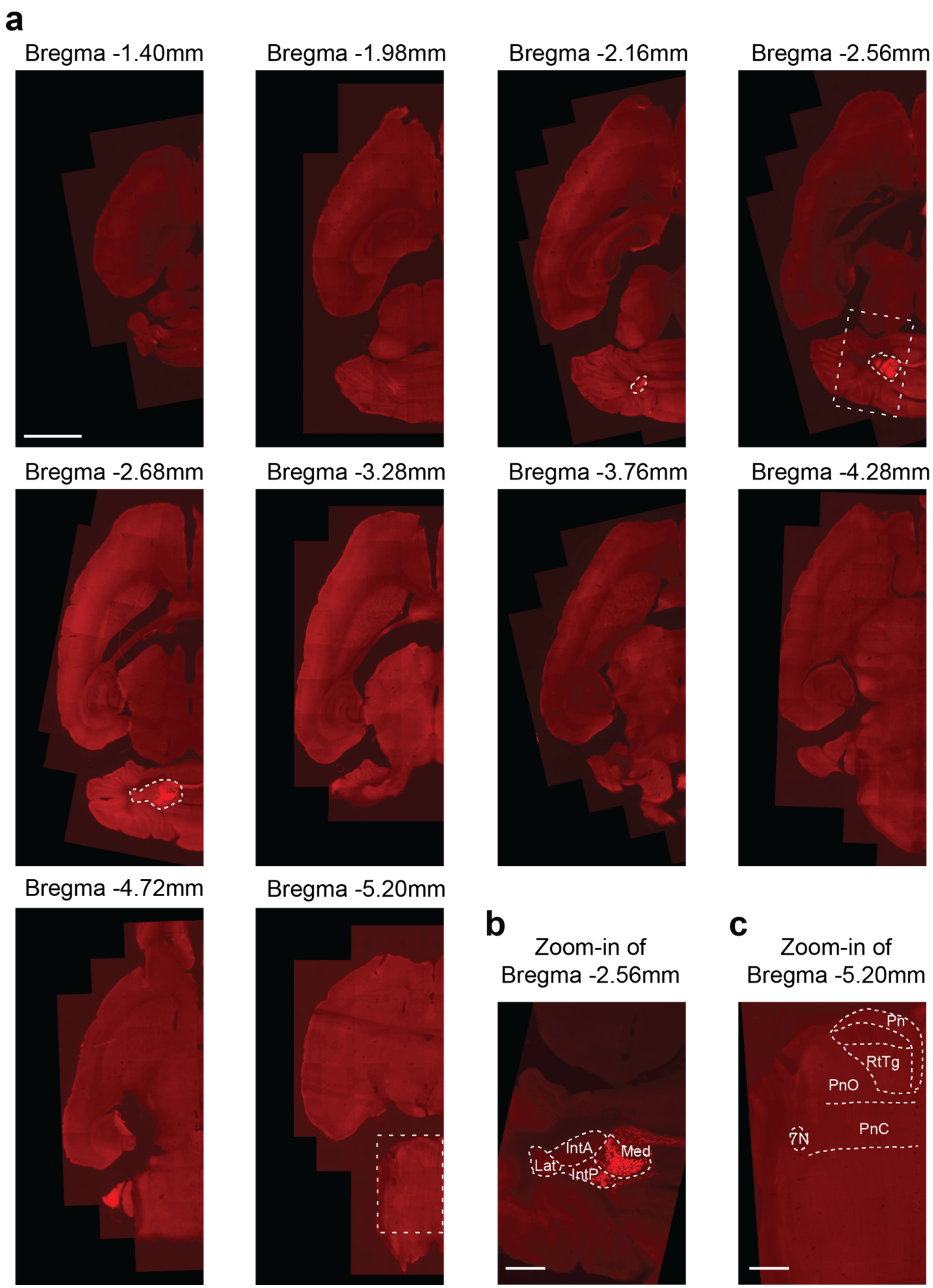

Supplementary Figure 5. Selectivity of viral DCN-targeting.

a, Whole-brain analysis of cells infected upon injection of AAV2-DiO-hM4Di-mCherry viruses into the medial and interposed deep cerebellar nuclei of vGluT2cre mice (as used in Figures 6 and 7, N=2 mice). Montages of tiled, high-resolution confocal images acquired for brain sections from indicated bregma levels. The cells exhibiting hM4Di-mCherry expression are confined to the medial DCN and posterior interposed nucleus. This analysis demonstrates that the AAV variant used in these experiments does not result in detectable retrograde infection of cell populations outside the DCN. Scale bar: 2mm.

b, Magnification of dashed area at bregma -2.56 (DCN). Scale bar: 500µm.

c, Magnification of the pontine gray nucleus (a major source of axons innervating the DCN).

Pn – pontine nucleus; PnO – pontine nucleus, oral part; PnC – pontine nucleus, caudal part; RtTg - reticulo tegmental nucleus; 7n – facial nerve. Scale bar: 500µm.

Supplementary Figure 6

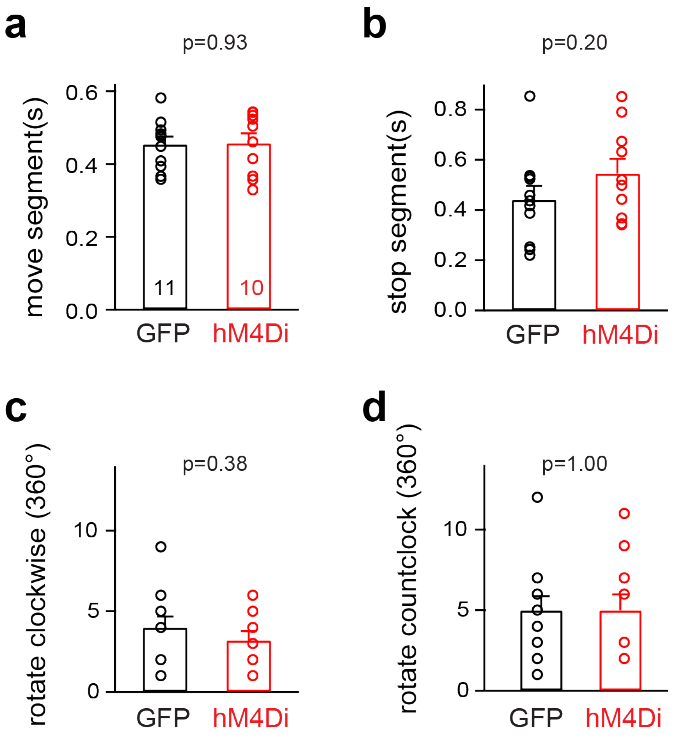

Supplementary Figure 6. Locomotion in open field arena of mice with silenced DCN neuron activity.

a, The average duration of movement bouts in CNO-treated DCN^{GFP} control and DCN^{hM4Di} mice was calculated by dividing the total moving time by the frequency of movement bouts (N=11 and 10 mice, respectively, mean±SEM, unpaired t-test).

b, The average duration of immobility segments was calculated by dividing the total non-moving time by the frequency of stopping events, (N=11 and 10 mice, respectively, mean±SEM, unpaired t-test).

c,d Clockwise and counter-clock wise rotations of 360° in CNO-treated DCN^{GFP} control and DCN^{hM4Di} mice (N=11 and 10 mice, respectively, mean±SEM, unpaired t-test).

Supplementary Figure 7

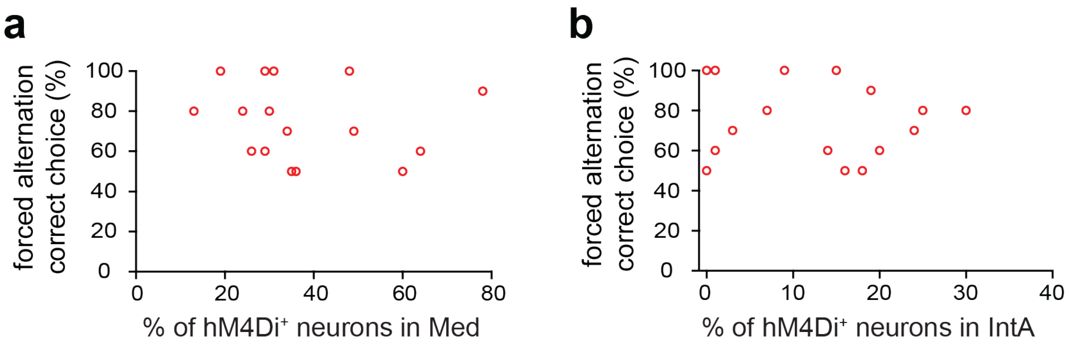

Supplementary Figure 7. Performance of mice with silenced DCN in the forced alternation test.

a,b, No significant correlation between the density of hM4Di-expressing cells in medial (Med in a) or Interposed anterior (IntA in b) deep cerebellar nuclei (N=16 mice). The rate of correct choices in the forced alternation test was plotted over the percentage of hM4Di expressing neurons (analysis done as in Fig. 7j for IntP).
